# Indian experience with immunotherapy in sarcoma and gastrointestinal stromal tumors: a retrospective study

**DOI:** 10.2144/fsoa-2021-0117

**Published:** 2022-04-20

**Authors:** Rohit Reddy, Raja Mounika Velagapudi, Sindhura Durga Chitikela, Adarsh Barwad, Shakti Shrivastava, Ekta Dhamija, Shamim Ahmed Shamim, Sarthak Tripathy, Rambha Pandey, Sameer Rastogi

**Affiliations:** 1 Department of Medical Oncology, Institute Rotary Cancer Hospital, All India Institute of Medical Sciences, New Delhi, 110029, India; 2 Department of Internal Medicine, Venkateshwara Institute of Medical Sciences, Uttar Pradesh, 244236, India; 3 Department of Pathology, Institute Rotary Cancer Hospital, All India Institute of Medical Sciences, New Delhi, 110029, India; 4 Department of Radio diagnosis, Institute Rotary Cancer Hospital, All India Institute of Medical Sciences, New Delhi, 110029, India; 5 Department of Nuclear Medicine, All India Institute of Medical Sciences, New Delhi, 110029, India; 6 Department of Radiation Oncology, Institute Rotary Cancer Hospital, All India Institute of Medical Sciences, New Delhi, 110029, India; 7 Sarcoma Medical Oncology Clinic, Department of Medical Oncology, Institute Rotary Cancer Hospital, All India Institute of Medical Sciences, New Delhi, 110029, India

**Keywords:** alveolar soft-part sarcoma, checkpoint inhibitors, disease control rate, GIST, immune-related toxicities, immunotherapy, sarcoma, undifferentiated pleomorphic sarcoma

## Abstract

**Aim:**

To study the role of check point inhibitors (CPI) in sarcoma and gastrointestinal stromal tumors.

**Materials & methods:**

Retrospective data of 15 patients diagnosed with advanced sarcoma or gastrointestinal stromal tumors and treated with CPI.

**Results:**

3/14 patients (21.4%) responded to treatment with a disease control rate of 42.8% (6/14). After a median follow-up of 14 months (range: 2–24 months), 11 (73.3%) patients progressed, the median progression-free survival was 4 months (95% CI: 1.7–6.3) and median overall survival was 14 months (95% CI: 2.6–25.7). Only one patient experienced a grade IV adverse event.

**Conclusion:**

Our data represent the first real-world application of CPI in sarcoma from India. We believe that CPI should be further evaluated in clinical trials.

## Plain language summary

Immunotherapy has led to durable responses in lung cancer and melanoma. Similarly, it is also being explored in sarcoma, a rare subtype of cancer, here we report data regarding immunotherapy in sarcoma and gastrointestinal stromal tumors at our institute [1]. A total of 15 patients were treated and clinical benefit was noted in 40% patients with two patients having a durable response. Side effects of the treatment are tolerable with only one patient experiencing a serious side event as pneumonia. Hence, we believe that immunotherapy has a role in sarcoma and should be explored in future studies.

In the last few years, immunotherapy has shown to be effective in malignancies such as lung, kidney, melanoma, bladder, etc. However, in soft-tissue sarcoma (STS) its role is not clearly defined and studies are ongoing. For immunotherapy to work in any cancer, it needs to be ‘hot’ or ‘inflamed’, in other words, highly immunogenic, with high proportion of tumor infiltrating lymphocytes (TILs) and tumor activating macrophages (TAMs) and actively expressing immune check points. D'Angelo *et al.* in their seminal paper studied immune profile in 50 patients of various subtypes of STS and they tried to find a correlation with biomarker expression and overall survival in their study [2]. Low immunogenic subtypes noted in the study (TILs <5%) were leiomyosarcoma (LMS), synovial sarcoma and chondrosarcoma and similarly gastrointestinal stromal tumors (GIST) had a high immunogenic potential (TILs >5%). The study could not establish any correlation with biomarker expression and overall survival.

Programmed death 1 (PD-1) and cytotoxic T-lymphocyte-associated antigen 4 (CTLA-4) are immune check points that negatively regulate T-cell immune function allowing the cancer cells to escape the host immune surveillance. Hence check point inhibitors (CPI) of these molecules have led to immunotherapies being employed for treatment of many cancers [3]. The initial evidence of efficacy of anti-PD-1 CPI in sarcoma comes from the SARC 028, a nonrandomized multi-cohort phase II trial conducted by Tawbi *et.al* [4]. In which 42 patients of different subtypes of STS were included and given pembrolizumab 200 mg every three weekly. With a median follow-up of 17.8 months, a total of 7/42 (18%) patients responded to therapy. The activity was limited to undifferentiated pleomorphic sarcoma (UPS) subtype with 4/10 (40%) patients responding, liposarcoma (LPS) subtype with 2/10 (20%) responders and only one synovial sarcoma patient responding to CPI and no responses observed among the leiomyosarcoma (LMS) subtype. Among the bony sarcomas, 2/40 (5%) patients had an objective response to CPI. On the other hand, monotherapy with CTLA-4 inhibitors was largely unsuccessful, ipilimumab was studied in the pilot study of six patients with advanced synovial sarcoma and the study was prematurely terminated because of no responses [5]. In a phase II study, presented at ASCO 2021 including patients with advanced ASPS (n = 44), atezolizumab showed a response rate of 37% (16/43) with one patient experiencing a complete response (CR) and 14 pts experiencing a partial response (PR) and 25 patients showing a stable disease [6]. However, not all sarcoma subtypes respond to CPI as exemplified by the phase II study by George *et al.* in which, single agent nivolumab was given in 12 patients with uterine LMS and none responded to treatment [7].

Taking a cue from melanoma the combination therapy of anti-PD1 and anti-CTLA 4, Alliance A091401 study [8], which was a phase II non-comparative, open-label trial randomized 85 patients of STS to nivolumab monotherapy or a combination of nivolumab with ipilimumab followed by maintenance nivolumab. The responses with single-agent nivolumab were 5 and 16% with the combination regimen.

In a phase II randomized study by *Singh* *et al.* [9] of 20 patients with advanced or metastatic GIST, post progression on at least imatinib, treatment with a combination of nivolumab or nivolumab with ipilimumab for 2 years, showed few responses. The median progression-free survival (PFS) in both the arms was 8 weeks. Thus, immunotherapy in GIST is still experimental and with availability of newer effective tyrosine kinase inhibitors (TKI) like ripretinib and avapritinib, its applicability is questionable.

Hence, it is clear from the above literature review that immunotherapy is effective in at least a subset of patients with sarcomas. However, most of the available literature of immunotherapy in sarcoma did not include the Indian and southeast Asian population. Hence, we conducted this retrospective review at a tertiary referral center in North India, to evaluate efficacy and tolerability of immunotherapy in our patients with advanced sarcoma.

## Materials & methods

The study was retrospective in nature. Patients included were advanced sarcoma and GIST patients treated with anti-PD-1 CPI from June 2017 to June 2021 in sarcoma medical oncology clinic at an Indian tertiary cancer referral center. All patients with advanced sarcoma treated with immunotherapy during this period were included in the study. All cases were reviewed by a dedicated sarcoma pathologist and discussed in a multidisciplinary clinic before starting immunotherapy. There was only one case of discrepancy between the initial and final diagnosis before starting CPI (patient no. 14). The data was collected from the hospital records.

Baseline characteristics analyzed included age, sex, site of disease and the location of metastasis, subtype of sarcoma, prior number of lines of treatment. Treatment-related details noted were dose and duration of immunotherapy, response rate and outcomes and toxicities of drugs. PD-L1 status was assessed by IHC and score was calculated based on the percentage of tumor cells showing distinct membrane positivity. PD-L1 status was assessed using DAKO PD-L1 immunohistochemistry (IHC) assay and tumor sample was deemed to be positive if IHC showed a membrane positivity in ≥1% tumor cells. This is finally reported as tumor proportion score (TPS). Facility for tumor mutational burden (TMB) estimation was not available in-house and microsatellite instability testing was not considered as the correlation with outcomes in STS was not deemed adequate. Patients were analyzed for response clinically during every visit and radiologically every two to three monthly. Response was evaluated based on RECIST 1.1 criteria radiologically either using computed tomography (CT) scan or magnetic resonance imaging (MRI) with positron emission tomography (PET/CT) used in ambiguous cases only. Disease control rate (DCR) was defined as a sum of complete response (CR) + partial response (PR) + stable disease (SD) at 3 months of therapy.

### Statistical analysis

The statistical analysis was done via SPSS 23. Baseline characteristics were analyzed using descriptive statistics. Nominal data were presented as number (%) and continuous data as median (range). Overall response rate (ORR) was calculated as a sum of complete response (CR) and partial response (PR) using RECIST 1.1. Data on patients who were progression-free were censored on the date the patient was last seen. PFS was calculated from the day of start of immunotherapy till the documentation of a progressive disease. OS was calculated from the date of start of CPI till death. PFS and OS curves were estimated with the Kaplan–Meier method. The entire population was assessed in terms of baseline characteristics and toxicity details but only in patients where the response evaluation to treatment was available (14/15 patients) were used for response and outcomes assessment.

## Results

Table 1 represents the baseline characteristics. A total of 15 patients were treated with anti-PD-1 CPI during the study duration. All of treated patients had metastatic disease when they received the agents. The median age of the patient population was 49 years (range: 18–71 years). Majority of the treated patients were male (n = 12; 80%). The histology of the primary disease treated is listed in Table 1. The median duration from the primary diagnosis till the start of immunotherapy was 27 months (range: 6–105 months). The most common sites of metastasis at the start of CPI were lung (n = 10; 66.6%) followed by bone (n = 6, 40%). Ten patients (n = 10; 66.6%) had more than two sites of metastasis and the average number of metastases were 12 reflecting the heavy burden of disease of our patients. Nine patients (60%), had a poor PS at start of anti PD1-CPI. The median number of prior lines of treatment were 2 (range: 0–6). These features describe the advanced nature of disease, post multiple treatments with limited further options of therapy. Our study had a total of 13 STS and 2 GIST patients, though both have different pathobiology and treatment modalities, at our institute we treat them under a common clinic and hence have included GIST patients.

**Table 1.** Base line characteristics of patient cohort.

Patient no.	Age (years)	Sex	Primary site	Histologic subtype	Site of metastasis	Prior lines of treatment (n)	ECOG PS
1	18	M	Neck	CCS	Lung, bone, nodal	2	1
2	54	M	Hand	ES	Lung, brain, nodal	3	2
3	31	M	Paravertebral	MPNST	Nonregional lymph nodes	5	3
4	49	M	Stomach	GIST	Liver, bone, omental	2	1
5	28	M	Stomach	GIST	Liver, pleural effusion, omental	4	1
6	26	M	Ankle	CCS	Nonregional lymph nodes	1	1
7	49	M	Leg	ASPS	Lung and lymph nodes	4	2
8	20	F	Buttock	ASPS	Lung, bone, subcutaneous	2	2
9	63	F	Thigh	UPS	Lung	2	1
10	50	M	RP	LMS	Liver, bone	6	2
11	72	F	RP	LPS	Bone, lung	1	2
12	71	M	Humerus	Dd CS	Lung	0	2
13	53	M	Thigh	UPS	Lung	3	2
14	47	M	RP	LMS	Lung, soft tissue, bone	5	1
15	18	M	Leg	ASPS	Lung	5	2

ASPS: Alveolar soft-part sarcoma; CCS: Clear-cell sarcoma; Dd CS: Dedifferentiated chondrosarcoma; ES: Epithelioid sarcoma; GIST: Gastrointestinal stromal tumor; LMS: Leiomyosarcoma; LPS: Liposarcoma; MPNST: Malignant peripheral nerve sheath tumor; Rp: Retroperitoneum; UPS: Undifferentiated pleomorphic sarcoma.

**Figure 1.**
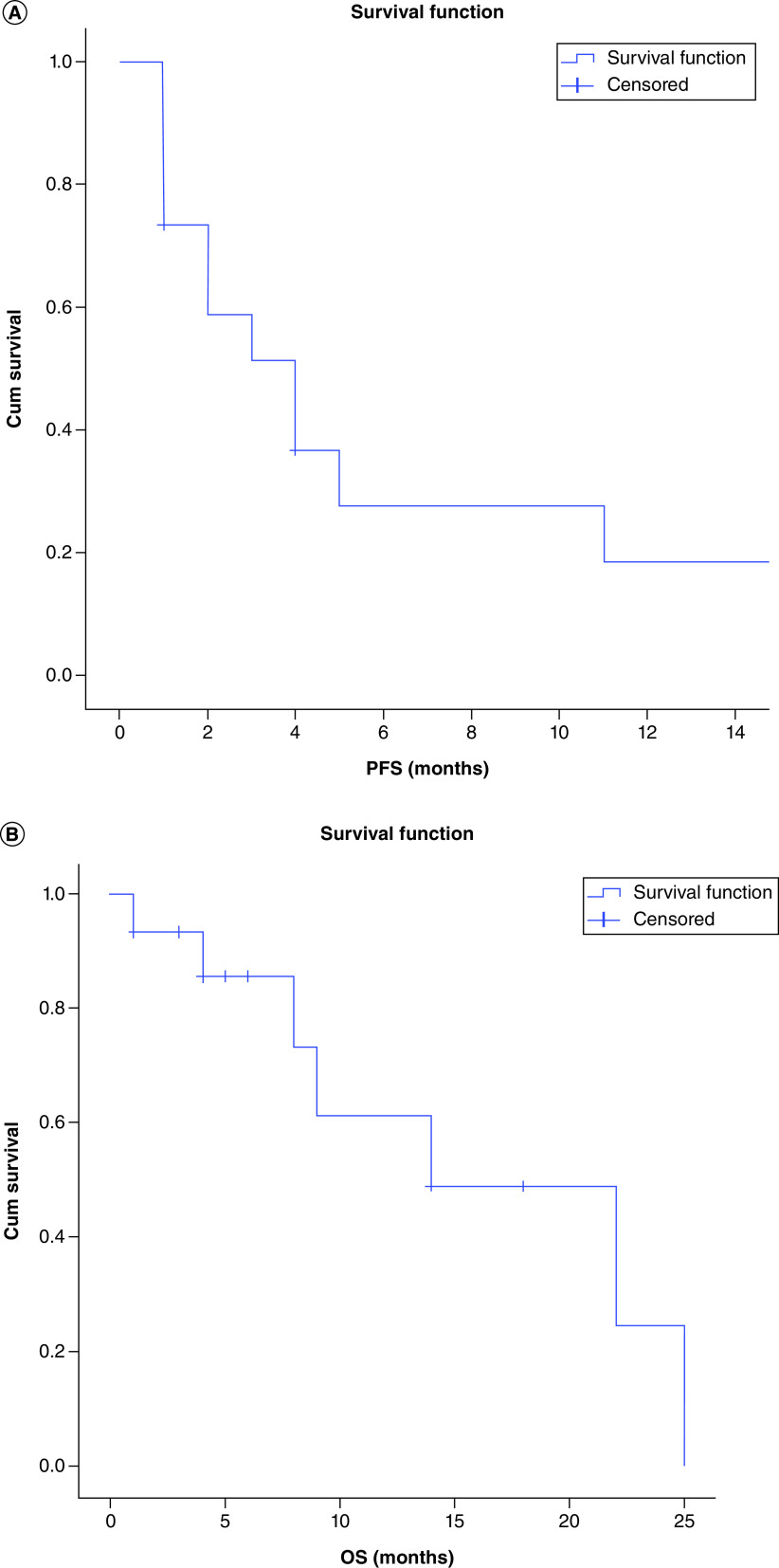
Progression free time in days grouped by best overall radiographic response in patients. Y-axis represents patient no., treatment used and diagnosis (in parenthesis), and x-axis represents the duration of response. Arrows indicate ongoing immunotherapy treatment at time of analysis (response evaluation of patient no. 15 pending at the time of final manuscript). Standard dosing used in all patients except patient no.7, where low dose nivolumab was used. ASPS: Alveolar soft-part sarcoma; CCS: Clear-cell sarcoma; CR: Complete response; Dd CS: Dedifferentiated chondrosarcoma: ES: Epitheloid sarcoma; GIST: Gastrointestinal stromal tumor; LMS: Leiomyosarcoma; LPS: Liposarcoma; MPNST: Malignant peripheral nerve sheath tumor; Nivo: Nivolumab; PD: Progressive disease; Pembro: Pembrolizumab; PR: Partial response; SD: Stable disease; UPS: Undifferentiated pleomorphic sarcoma.

**Figure 2.**
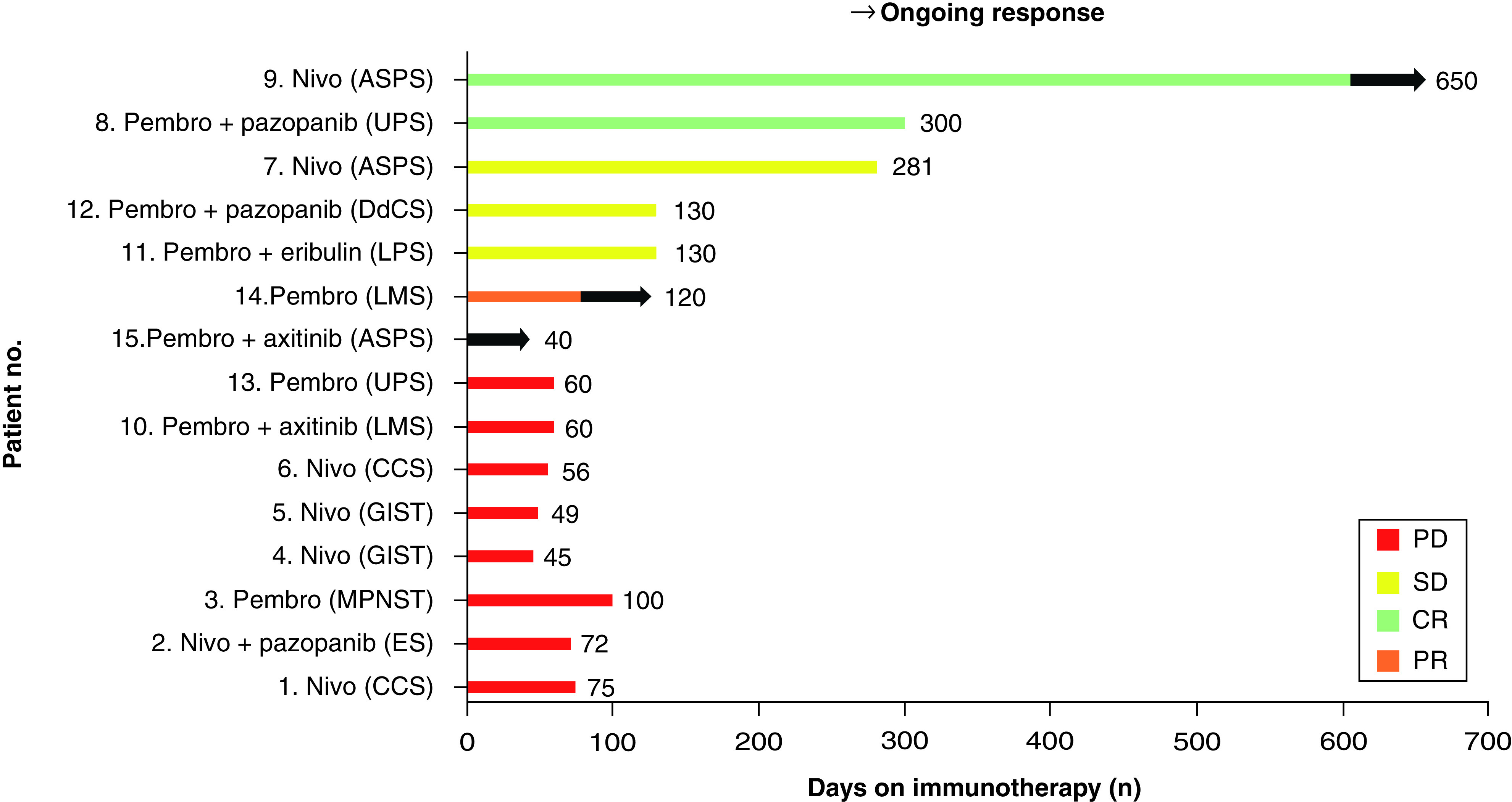
Progression free survival and overall survival using immunotherapy. OS: Overall survival; PFS: Progression-free survail.

Table 2 represents the treatment details of study population, anti-PD-1 CPI utilized were nivolumab, in seven patients (n = 7; 43.7%) and pembrolizumab in eight (n = 8; 56.7%) patients, combination treatment with TKI was used in five patients (33.3%). Combination of CPI with chemotherapy was used in one patient. Standard dosing of immunotherapy was used in all patients, except for one patient (patient no. 7) where a lesser dose was used because of financial constraints. Overall response rate (ORR) of the study population was 21.4% (3/14) with two patients achieving CR (14.2%) and one patient achieving PR (7.1%) as per RECIST 1.1 criteria. Three patients had SD (21.4%) and hence disease control rate (DCR) was 42.8%. Since none of the GIST patients responded to the treatment and considering STS cohort alone (13 patients), with response available in 12 patients, ORR was 3/12 (25%) and DCR was 6/12 (50%), respectively. Duration of therapy and details of response using CPI of all patients is depicted in Figure 1. After a median follow-up of 14 months (95% C.I 0 to 34.7 months). Median progression free survival (PFS) was of treated population was 4 months (95% CI: 1.7–6.3 months) (Figure 2). PD-L1 IHC status was available in 10 (66.6%) patients, and four patients had a positive PD-L1 positivity (TPS ≥1%). At the time of reporting data, 11 patients (73.3%) discontinued treatment, 10 patients (66.6%) experienced a progressive disease (PD), whereas one patient stopped treatment due to grade IV immune-related pneumonitis. Eight patients have died during study (53.3%) due to PD. Median OS was 14 months (95% CI: 2.6–25.7 months) (Figure 2). Anti-PD-1 CPI were well tolerated, with adverse events of all grades, noted in six patients (40%). Most common adverse event noted was hypothyroidism in three patients (20%). Serious adverse event leading to treatment discontinuation was noted in one patient who developed grade 4 immune related pneumonitis. No treatment-related deaths were noted in study.

**Table 2.** Patient treatment details with best responses.

Serial no.	CPI used	PD-L1 IHC (%)	Duration of therapy (months)	Dosage and frequency	Best response	Toxicities
1	Nivolumab	0	3	3 mg/kg q 2 weekly	PD	None
2	Nivolumab + Pazopanib	10	3	3 mg/kg q 2 weekly	PD	None
3	Pembrolizumab	-	2	200 mg q 3 weekly	PD	None
4	Nivolumab	0	2	3 mg/kg q 2 weekly	PD	None
5	Nivolumab	80	2	3 mg/kg q 2 weekly	PD	None
6	Nivolumab	-	3	3 mg/kg q 2 weekly	PD	None
7	Nivolumab	-	8	0.7 mg/kg q 2 weekly	SD	Grade II hypothyroidism
8	Nivolumab	0	24	3 mg/kg q 2 weekly	CR	Grade II hypothyroidism, fever
9	Pembrolizumab + Pazopanib	25	13	200 mg q 3 weekly	CR	Grade II hypothyroidism, grade IV pulmonary toxicity
10	Pembrolizumab + Axitinib	0	3	200 mg q 3 weekly	PD	Grade II dermatitis
11	Pembrolizumab + Eribulin	-	3	200 mg q 3 weekly	SD	None
12	Pembrolizumab + Pazopanib	0	3	200 mg q 3 weekly	SD	Grade II vitiligo
13	Pembrolizumab	0	5	200 mg q 3 weekly	PD	None
14	Pembrolizumab	1	3	200 mg q 3 weekly	PR	None
15	Pembrolizumab + Axitinib	-	1	200 mg q 3 weekly		None

IHC: Immunohistochemistry; PD: Progressive disease; PR: Partial response; SD: Stable disease.

## Discussion

Data regarding CPI in advanced soft-tissue and bony sarcomas is largely available from the Western literature. The reasons for lack of data of immunotherapy from developing world countries stems from conspicuous lack of clinical trials with anti PD1 CPI in rare cancers like sarcoma and GIST. Hence, we conducted this retrospective study to obtain an insight into the role of anti PD1-CPI in sarcoma.

Median age in our patients was 49 years as compared with SARC 028 trial where it was 53 years, indicating the younger population cohort of soft-tissue sarcoma (STS) noted in Indian patients [10]. The limited data regarding the subtypes of sarcoma of Asia population appears similar to the Western literature with most common subtypes being LPS, UPS and LMS, however the data regarding the incidence of other subtypes such as ASPS, CCS are limited as they are rare [11]. The study also had a higher proportion of male patients compared with other studies on immunotherapy [4], correlating with the predominant male population of STS seen in India. Study population represents the real-world scenario of sarcoma patients who had a high burden of advanced disease, reflected by median number of sites of metastasis being two (range: 1–3), average number of metastases in each patient of 12. Nine patients (58.3%) had poor PS, ECOG PS ≥2. Median number of prior treatment regimens were two (range: 0–6). The median duration for start of immunotherapy from the point of diagnosis of sarcoma was 27 months (range: 6–108 months). This indicates the advanced and chemo refractory nature of disease in this patient cohort, where in anti-PD-1 CPI were considered as a desperate option, very late in their clinical course and because it was not a clinical trial, dose used was uniform in all patients. This is unlike studies on CPI in advanced sarcoma conducted in the West, where treated patients had usually a good PS, and were treated relatively early in the disease course [4].

Three patients (21.4%) had objective clinical response to treatment with two patients achieving CR. Patient no. 8 and 9 with a diagnosis of UPS and LMS achieved CR with CPI and patient no. 14 with a primary diagnosis of LMS achieved a PR with CPI. The disease control rate (DCR) was 42.8% in study population. Although these patients were chemo-refractory, the observed results are encouraging with a median PFS of 4 months in entire cohort including all 14 patients. The median PFS in our study might be slightly lower than other studies as the patients had advanced, chemo refractory disease with a heavy burden of disease and poor PS unlike the Western studies [8]. We believe that the benefit of CPI in sarcoma cannot be based on median PFS or ORR alone which is similar to CPI responses noted in melanoma, though the median PFS of CPI and chemotherapy differ by only 2 months, duration of response is much higher with CPI as compared with chemotherapy [12], however this hypothesis has to be validated prospectively.

**Figure 3.**
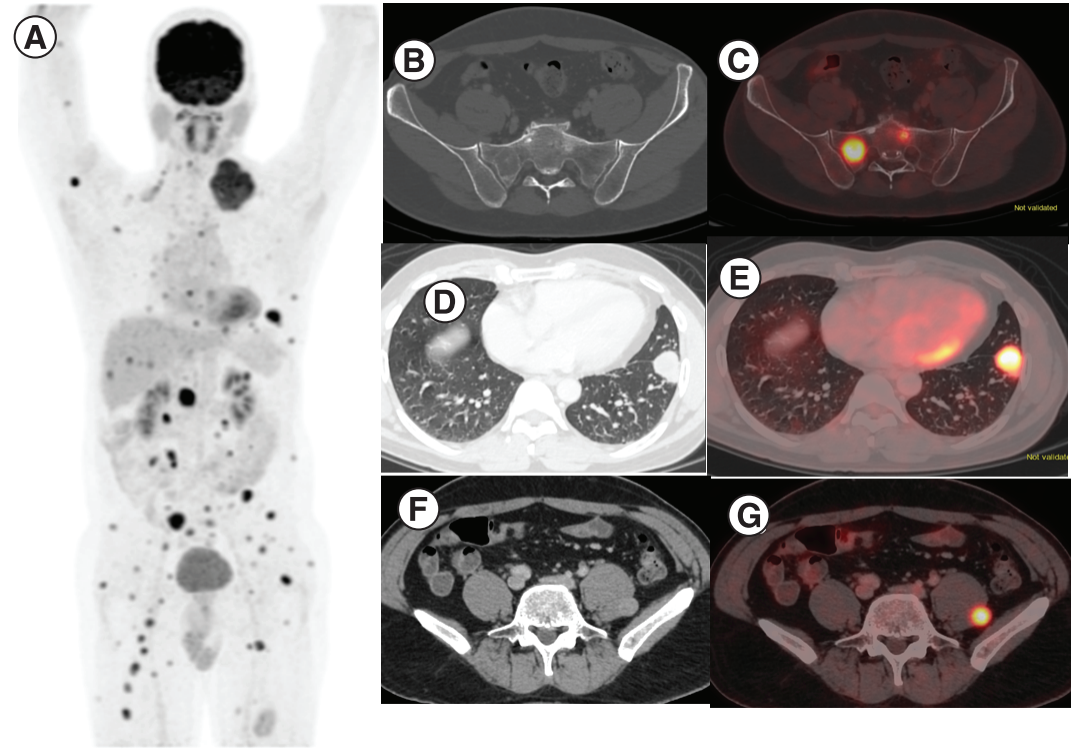
Pre- and post-treatment images of patient no. 9. **(A)** Pre-treatment axial contrast-enhanced computed tomography thorax showing soft-tissue density in left upper lobe apico-posterior segment. **(B)** Corresponding fused positron emission tomography-computed tomography image showing increased tracer uptake suggesting lung metastasis. **(C & D)** Represent post-therapy changes in same patient with complete resolution of metastasis with fibro reticular changes s/o complete response with residual drug toxicity changes.

**Figure 4.**
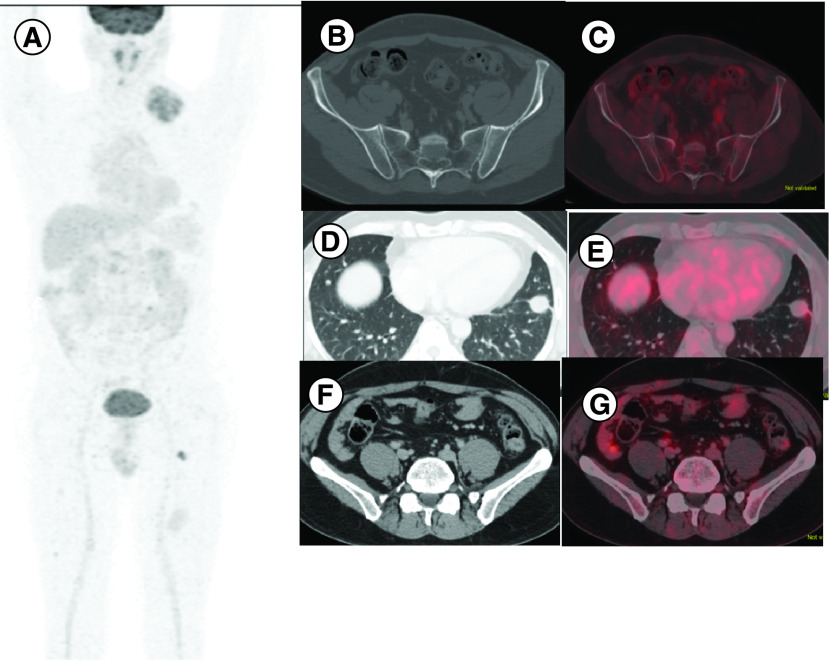
Axial contrast-enhanced computed tomography thorax of patient no. 9 showing bilateral lower lobe fibro consolidatory changes with fused positron emission tomography-computed tomography image showing mild tracer uptake suggesting drug-related changes.

Role of immunotherapy in sarcoma appears to be histology specific, its activity appears be relatively better in subtypes like UPS, ASPS, LPS. Vascular endothelial growth factor (VEGF) promotes immunosuppressive microenvironment [13]. Anti VEGF TKI may reverse this phenomenon. Hence a combination of TKI with anti PD 1-CPI is postulated to have synergistic effects. In IMMUNOSARC study, a combination of sunitinib (TKI) and nivolumab were used and good clinical benefit was across all the subset of sarcoma, and 50% patients remained progression free at 6 months [14]. Five patients (33.3%) in our study had received a combination of immunotherapy with TKI. Patient no. 9 with UPS had PR to the combination of pembrolizumab with pazopanib at 3 months of therapy [15]. The same patient developed CR after continued treatment with anti-PD-1 CPI alone (Figure 3). She developed grade IV pneumonitis while on treatment and the drug had to be discontinued (Figure 4). Interestingly even after discontinuing treatment she continued to be in disease remission. In the final expansion cohort of SARC 028 trial, published in ASCO 2019, UPS cohort had the best response, response rate of 23% (9/40) [16].

ASPS is an indolent chemo resistant tumor, however anti VEGF TKI play an important role in treatment [17]. ASPS is also an immunotherapy sensitive subtype, as the data of CPI in ASPS is more robust, many of our patients with ASPS received CPI early in the disease course. A total of three ASPS patients were started on CPI, two patients had clinical benefit, one patient achieved CR, the other despite using a lower dosage of drug, had SD for 8 months, the response of the last patient on CPI is pending. Wilky *et al.* conducted a phase II study [18], single arm study in 33 patients of STS. The cohort had 36% patients (n = 12) with ASPS. With a median follow-up of 14.7 months, 3-month PFS for overall study patients was 65.6% (95% CI: 46.6–79.3). For ASPS subtype, 3-month PFS was 72.7% (95% CI: 37.1–90.3). Median PFS was 12.4 months in patients with ASPS subtype compared with 3.0 months in others. Recently low dose nivolumab (20 mg–100 mg q 2 weekly) has been tried in non-small cell lung cancer [19]. We similarly used low dose nivolumab in our patient based on this data.

**Figure 5.**
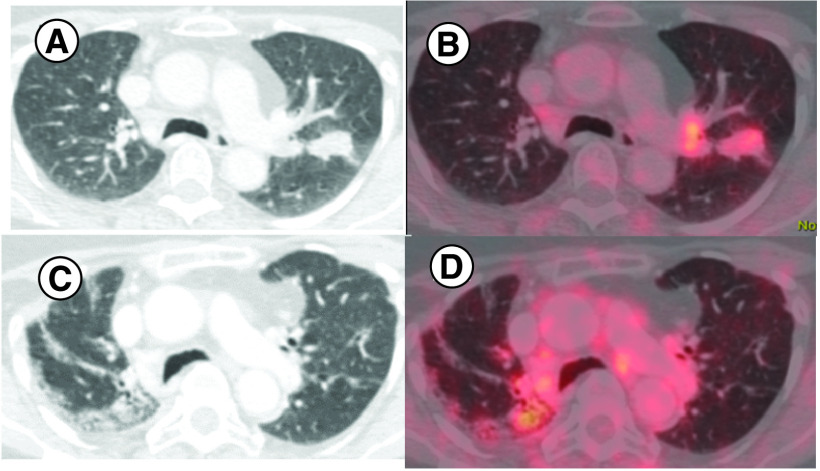
Pre treatment images of patient no.14, case of leiomyosarcoma. **(A)** Maximum intensity projection image of fused fluorodeoxyglucose positron emission tomography with computed tomogram (FDG PET-CT) of patient no.14 showing multiple areas of tracer uptake. **(B)** Axial CT images shows lytic skeletal lesions. **(C)** Shows increased FDG uptake in corresponding fused PET-CT. **(D)** Lung metastases in axial CT images. **(E)** Lung metastases with FDG uptake in fused PET-CT. **(F)** Intramuscular deposits in axial CT images. **(G)** FDG uptake in fused PET-CT image .

**Figure 6.**
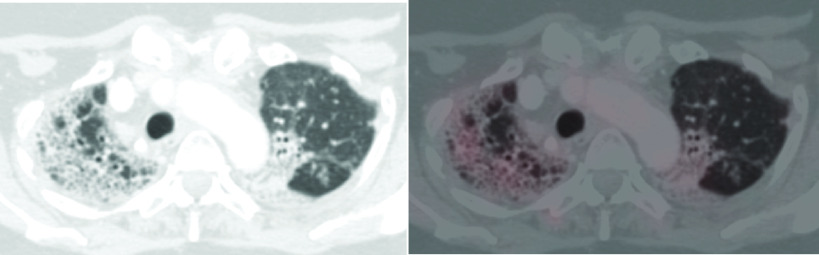
Post treatment images of patient no. 14, case of leiomyosarcoma. **(A)** Maximum intensity projection image of fused fluorodeoxyglucose positron emission tomography with computed tomogram (FDG PET-CT) of patient no. 14 showing decreased areas of tracer uptake. **(B)** Resolution of lytic skeletal lesions in axial contrast-enhanced computed tomography images. **(C)** No FDG uptake in fused PET-CT. **(D)** Lung metastases. **(E)** Mild FDG uptake in fused PET-CT. **(F)** Resolution of intramuscular deposits. **(G)** No FDG uptake in fused PET-CT image. Overall findings suggestive of partial response.

Leiomyosarcoma is usually characterized by high TILs and PD-L1 expression [2]. Many studies on CPI in sarcoma have classically shown that LMS subtype doesn't show any clinical response to anti PD-L1 CPI [8,20]. But there are occasional case reports where anti PD-L1 CPI has shown excellent clinical response with prolonged disease remission in LMS [21]. Similarly, we tried anti PD-L1 CPI in two of our patients with LMS of which one patient had objective response as PR (Figure 5,6). Such results are encouraging; and we recommend further research in exploring role of CPI in LMS.

Anthracyclines form the first line treatment option in most of the metastatic soft-tissue sarcomas. It is hypothesized that combination of cytotoxic chemotherapy with anti PD 1-CPI, may improve its efficacy by depleting immunosuppressive cells and releasing damage-associated molecular patterns (DAMP) [22]. The combination of doxorubicin with pembrolizumab was explored in recent phase I/II study by Pollack *et al.*, and they noted responses in UPS and LPS subsets. Responses were also noted in chondrosarcoma including one patient with conventional chondrosarcoma which is classically described as chemo resistant tumor [20]. Similarly, a phase II study of pembrolizumab with eribulin in metastatic sarcomas is ongoing, the initial results reported of the LMS cohort have not met the predetermined end point, data of LPS cohort is still pending [23]. As the benefit of CPI in LPS subtype is not clear yet, we had treated our patient with LPS, who had progressed on a prior anthracycline regimen with a combination of eribulin and pembrolizumab and was progression-free for four months with CPI. We also treated a patient with dedifferentiated chondrosarcoma with combination of pembrolizumab with pazopanib who also experienced good disease control with SD for 3 months (Figure 1).

Not all subtypes of STS show response to CPI. Other than isolated case reports of efficacy of immunotherapy in malignant peripheral nerve sheath tumor (MPNSTs) [24], major studies of immunotherapy have been largely unsuccessful [6]. Even our patient with MPNST, has failed treatment with of immunotherapy. Similarly though study by Singh *et al.* on patients with advanced GIST [9], showed some response to CPIs, both our patients have progressed on immunotherapy even though one of them had high PDL1 expression, TPS = 80%, hence reiterating the fact that response to CPI in sarcoma may be histology specific and that no conclusive biomarker has yet been identified so far which can predict response to immunotherapy in sarcoma. Though one might argue that GIST is a different disease, which is unlike sarcoma, at our institute we treat them under a common clinic and hence we wanted to highlight our experience with CPI in GIST. In the retrospective cohort of 11 patients, treated with immunotherapy in clear-cell sarcoma [25], final analysis did not show benefit of immunotherapy in comparison to standard chemotherapy. Two patients with clear-cell sarcoma in our cohort did not respond to immunotherapy. In epithelioid sarcoma, which is a very aggressive disease with dismal outcomes, immunotherapy has been tried but largely unsuccessful with only occasional responses [26]. The patient, whom we had started on immunotherapy has also failed treatment.

The role of biomarkers such as PD-L1 staining and correlation with response to immunotherapy is established in other malignancies [27]. Though sarcomas are considered highly immunogenic with studies showing a PD-L1 positive rates of 30–40%, the correlation with response rate to CPI has not yet been proven. In our study we had four patients with a positive PD-L1 (PD-L1 IHC >1%) but did not find a significant correlation with biomarker expression and response to treatment. In SARC028 trial, only 4% (3/70) of tumors samples had a positive stain for PD-L1 (TPS >1%), all three of them were UPS of which two patient responded to the treatment [4]. It is the only subset where positive correlation with biomarker expression was noted. Positive correlation with biomarker expression in our study was also seen in UPS subtype where one patient with high PD-L1 expression (patient no. 9) had complete response to anti PD-L1 CPI and the other patient with negative marker expression (patient no. 13) progressed on treatment. We did not notice a similar correlation in other subtypes as illustrated by patient no. 5, case of GIST with high PD-L1 expression but no response to therapy and other patient with ASPS (patient no. 8) with no expression but excellent response to treatment. Even other studies on ASPS subtype show a poor correlation between biomarker expression and response to therapy [18]. Hence correlation of biomarker expression with response to CPI might also be histology specific but since our study was retrospective cohort with a small sample size, we would recommend further research before making a conclusive statement. Tumor mutational burden was not performed as it was expensive and was not available in house. Mismatch repair deficiency testing was not performed as the data was not convincing regarding its role in sarcoma.

The adverse effect profile of patients treated with anti PD 1-CPI was favorable, most events were grade 1/2 in nature. The most common adverse event was hypothyroidism in three patients (25%). The other grade 1/2 toxicities noted were dermatitis, vitiligo and fever. One patient developed life-threatening grade 4 immune-related pneumonitis requiring intensive care support with permanent discontinuation of treatment. However, the same patient continues to be disease free even post 4 months after stopping treatment with anti-PD-1 CPI. No treatment-related deaths were noted in study. The adverse event rate appeared similar to other studies [28,29].

Our study limitations are that we have a small cohort size, which limited our ability to define a potential benefit with individual checkpoint inhibitors. It is also a retrospective series with its inherent biases. STS subtypes were heterogenous, with benefit predominantly been seen in a few subtypes. Dosing of CPI was not uniform which might have undermined potential clinical benefit. Similarly, the small sample size undermined our effort in finding specific histological subtypes of STS with response to immunotherapy. We also acknowledge that most of the patients were treated with CPI at time points when the data was upcoming with further advances in the treatment aspect further enabled us to precisely use the treatment. The role of biomarkers was not fully explored.

Indian patients are underrepresented in trials of CPI in sarcoma and our study represents the real-world data of CPI in sarcoma. We believe that in future multicentric studies, Indian patients should also be included in clinical trials.

## Conclusion

The results of our study are consistent with other studies. Our study represents the first attempt in India at exploring role of immunotherapy in sarcoma. Though it was a small cohort we had meaningful clinical responses in our patients who otherwise had progressive disease with standard treatment modalities. Learning from our experience, we suggest exploring immunotherapy in sarcoma as it can have meaningful and long-lasting clinical response in these patients.

## Future perspective

Utility of CPI in sarcoma is evolving. We believe in the coming years it could make an impact at least in certain subtypes such as ASPS and UPS. Hopefully trials could include Indian population for better applicability to our country.

**Summary points**

- Role of immunotherapy in soft-tissue sarcoma (STS) is slowly establishing itself yet currently it is still at an experimental stage.
- Unlike other cancers such as lung, bladder, colon, correlation of outcomes with PD-L1 expression is not yet clear in sarcoma. There is also no correlation with regards to biomarker expression and response to check point inhibitors (CPI). However certain subtypes such as alveolar soft-part sarcoma and undifferentiated pleomorphic sarcoma are more sensitive to CPI.
- With SARC 028, interest in the role of CPI in sarcomas was ignited.
- Our study was retrospective in nature, we treated 15 patients of advanced sarcomas and similarly gastrointestinal stromal tumors from June 2017 to June 2021 with CPI. All of them were treated with multiple lines of prior treatment.
- PD-L1 IHC was positive in 4 of 10 patients who were tested.
- Overall response rate was 20% and disease control rate (complete response + partial response + stable disease) was 42.7%.
- Median progression-free survival was 4 months.
- The treatment was tolerable with only one patient experiencing a grade IV pneumonitis.
- Our survival results correlate with available Western literature.
- We feel that role of CPI in sarcomas is evolving and hence recommend to explore CPI in sarcomas in a randomized trial. Trials should also include Asian population.
